# BoviFusionNet: A Lightweight Edge-Deployable AI System for Cattle Behavior Recognition in Livestock Monitoring

**DOI:** 10.3390/vetsci13070697

**Published:** 2026-07-17

**Authors:** Jiawen Li, Weidong Zhang, Ximing Ren, Jiarui He, Leijun Wang, Jujian Lv, Kaihan Lin, Wencai Du, Rongjun Chen

**Affiliations:** 1 School of Computer Science, Guangdong Polytechnic Normal University, Guangzhou 510665, China; lijiawen@gpnu.edu.cn (J.L.); zwd@stu.gpnu.edu.cn (W.Z.); renximing@gpnu.edu.cn (X.R.); hejiarui@stu.gpnu.edu.cn (J.H.); wangleijun@gpnu.edu.cn (L.W.); jujianlv@gpnu.edu.cn (J.L.); kaihanlin@gpnu.edu.cn (K.L.); 2 State Key Laboratory for Novel Software Technology, Nanjing University, Nanjing 210023, China; 3 ZUMRI-LYG Joint Laboratory, Zhuhai UM Science and Technology Research Institute, Zhuhai 519031, China; 4 Institute for Data Engineering and Science, University of Saint Joseph, Macau 999078, China; george.du@usj.edu.mo; 5 Guangdong Provincial Key Laboratory of Intellectual Property and Big Data, Guangdong Polytechnic Normal University, Guangzhou 510665, China

**Keywords:** cattle behavior recognition, livestock monitoring, edge AI, lightweight model, precision agriculture

## Abstract

This study aims to develop a lightweight, edge-deployable artificial intelligence (AI) system for real-time, non-contact recognition of cattle eating, standing, and lying behaviors in farm environments. Automated monitoring of these behaviors in cattle provides fundamental behavioral data for the future development of systems that analyze feeding duration, lying duration, and behavioral rhythms. Nevertheless, practical deployment on farms is hindered by data imbalance, dense animal groupings, scale variation, occlusion, and the need for low-cost edge computing. To address these challenges, we propose BoviFusionNet, a lightweight, edge-deployable AI system. A box balanced augmentation strategy rebalances training instances at the object level without altering the validation or test sets. Built upon YOLO11n, the model integrates three targeted enhancements: information-preserving downsampling (ADown), adaptive bidirectional feature fusion (BiFPN), and local window attention (C2CGA) to improve multi-scale representation and fine-grained behavior discrimination. Experimental results show that BoviFusionNet achieves 0.7851 recall, 0.7763 F1-score, 0.7976 mAP@0.50, and 0.6305 mAP@0.50:0.95, with only 5.4 GFLOPs and a 3.4 MB model size. Compared with the YOLO11n baseline, it improves mAP@0.50:0.95 by 9.92% and reduces the parameter count by 39.8%. After INT8 quantization and deployment on an RK3588S edge device, real-time inference reaches 28.08 frames per second (FPS). Therefore, BoviFusionNet offers an effective accuracy-complexity trade-off for on-farm edge AI applications. By enabling continuous, non-invasive monitoring of health-relevant behaviors, it provides fundamental behavioral data for the future development of veterinary health assessment tools without relying on cloud services or wearable sensors.

## Simple Summary

Farmers and veterinarians need to know how cattle eat, stand, or lie because changes in these behaviors can indicate potential health or welfare issues. Watching animals continuously is impossible on large farms, and wearable sensors can be expensive or stressful for the animals. In this study, we built a lightweight Artificial Intelligence (AI) system called BoviFusionNet that uses ordinary farm cameras to automatically recognize these three key behaviors in real time. This system is designed to run on small, low-power edge computers, avoiding the need for cloud servers or internet connections. To ensure the AI learns equally well from all behavior types, we developed a box-balanced training method that corrects data imbalances. When tested, our model achieved high accuracy while using very little memory and processing power. We then deployed it on a low-cost edge device (RK3588S), where it ran at 28 frames per second (FPS), fast enough for real-time monitoring. This practical, affordable AI system can provide foundational behavioral data to support the future development of systems that analyze eating duration, lying duration, and behavioral rhythms, which may ultimately enable earlier detection of health or welfare problems.

## 1. Introduction

Precision livestock farming is increasingly transforming cattle management from periodic manual inspection toward continuous, automated, and data-driven monitoring. Among various technologies, computer vision-based approaches offer a non-contact, cost-effective solution for monitoring group-level behavioral states without attaching sensors to individual animals [1]. In cattle production, eating, standing, and lying are fundamental behaviors closely linked to eating activity, welfare assessment, health status, and the early detection of abnormalities [2]. Therefore, reliable recognition of such behaviors can provide valuable information for daily farm management and for supporting veterinary decision-making, aligning with the central focus of artificial intelligence (AI) applications in livestock health and welfare.

Despite its practical value, computer vision-based detection of cattle behavior remains challenging in real barn and pasture environments. Cattle often appear in dense groups, suffer from partial occlusion, and show large-scale variations due to different distances from the camera [3]. In addition, behavior categories are not always visually separable by object appearance alone [4]. For example, eating, standing, and lying may share similar body textures and background conditions, while their discriminative cues are often reflected in local posture, head position, body contour, and surrounding context [5]. These challenges require an AI-based detector that can preserve spatial details, integrate multi-scale information, and enhance behavior-sensitive feature representation [6], all of which are essential for translating automated behavior monitoring into reliable inputs for health and welfare assessment.

Recently, several studies have developed methods for monitoring cattle behavior using You Only Look Once (YOLO)-based models [7,8,9], transformer-based models [10,11,12], pose estimation [13], and tracking-by-detection approaches [14]. Although these studies have demonstrated the feasibility of automated cattle monitoring, three key issues limit their practical application, particularly for real-time assessment in farm management and veterinary practice. First, cattle behavior datasets are often small and exhibit object-level imbalance. Unlike image classification, where each image provides a single label, object detection relies on annotated instances, in which a single image may contain multiple cattle and several behavior categories. Consequently, balancing only at the image level fails to supply balanced supervision for each behavior, potentially biasing the model toward more frequent categories. This imbalance can mask early signs of health disorders, such as reduced eating time or altered lying patterns. Second, high-capacity models such as two-stage detectors and transformer-based detectors can improve detection accuracy. Nevertheless, their computational cost and memory requirements make them unsuitable for low-power, low-cost edge devices, which are preferred for on-farm deployment to ensure data privacy, low latency, and cost-effectiveness. Third, compact one-stage detectors are more efficient, but they often lose accuracy in challenging scenarios involving dense crowds, occlusion, small targets, or visually similar behaviors, precisely the conditions that cause missed behavioral indicators of lameness, illness, or distress.

To address the above challenges, we propose BoviFusionNet, an AI edge-oriented lightweight system with box-balanced learning for real-time cattle behavior recognition. In detail, an object-level box-balanced offline augmentation method is used for the training split. Instead of simply increasing the number of images, this approach balances the number of annotated behavior instances among eating, standing, and lying. The validation and test splits are kept unchanged to maintain a clean and reproducible evaluation protocol. It improves supervision of instances of underrepresented behavior while avoiding contamination of the evaluation set, thereby supporting more equitable AI-based assessment and reducing the risk of diagnostic bias in veterinary applications.

Subsequently, BoviFusionNet is developed from a compact YOLO11-based model by employing three task-oriented components. Specifically, Adaptive Downsampling (ADown) is adopted in the backbone to reduce the loss of posture and boundary information during spatial downsampling. Bi-Directional Feature Pyramid Network (BiFPN) is used in the neck to perform learnable bidirectional multi-scale feature fusion, allowing the model to adaptively combine high-resolution spatial details with low-resolution semantic features [15]. Cross-Channel Cross-Guided Attention (C2CGA) is placed in the deep semantic stage to strengthen local window contextual modeling and improve fine-grained behavior discrimination, drawing inspiration from cascaded group attention for efficient visual representation [16]. Rather than simply enlarging the model, BoviFusionNet focuses on three key bottlenecks in cattle behavior recognition: (1) information loss during downsampling that weakens posture and boundary cues, (2) insufficient multi-scale fusion for dense and occluded cattle, and (3) weak fine-grained discrimination between visually similar behaviors. This task-specific integration is the core methodological novelty, as each component is designed to address a distinct practical challenge rather than being a generic architectural modification. These issues directly affect the accurate monitoring of health-relevant behaviors and, consequently, the timely assessment of health by farm veterinarians.

Lastly, to evaluate practical applicability, the proposed method is deployed on an RK3588S edge device, where edge AI deployment is beneficial for farm monitoring because cloud processing usually incurs additional latency, bandwidth consumption, and privacy concerns [17]. The embedded inference speed is determined not only by the parameter count or Giga Floating-Point Operations Per Second (GFLOPs) but also by operator compatibility, memory access patterns, quantization behavior, Neural Processing Unit (NPU) execution efficiency, and so on [18]. Accordingly, we report both server-side and board-side detection performance of the proposed framework, with a focus on enabling practical, low-latency, and privacy-preserving behavior monitoring on edge devices. Such edge-based, real-time detection directly empowers veterinarians and farmers to receive immediate alerts when behavioral deviations occur, facilitating early intervention, a cornerstone of preventive veterinary medicine. In short, the main contributions of this study are summarized as follows:

1. A box-balanced dataset of cattle behavior is constructed to recognize eating, standing, and lying behaviors. The training split is augmented according to the object-level distribution of behavior instances, using a source image selection strategy that minimizes instance-count gaps across classes. The validation and test splits remain unchanged. This dataset provides balanced supervision for each behavior category, addressing the challenge of information loss during downsampling that disproportionately affects underrepresented classes.
2. A lightweight edge-oriented AI system, BoviFusionNet, is proposed that integrates ADown, BiFPN, and C2CGA in a task-specific manner to address three bottlenecks identified in real cattle behavior monitoring: preserving posture information during downsampling, adaptive multi-scale feature fusion for dense/occluded cattle, and local window attention for fine-grained behavior discrimination. This integrated design improves cattle behavior recognition under dense, occluded, and multi-scale conditions prevalent in commercial farm environments.
3. Comprehensive experiments on the deployment of RK3588S are conducted. BoviFusionNet improves detection accuracy while reducing model complexity, and the INT8 RKNN deployment results demonstrate real-time feasibility for edge-side cattle behavior recognition. The results confirm that task-specific architectural integration, rather than indiscriminate model enlargement, is the key to achieving both accuracy and efficiency for practical edge-AI cattle behavior monitoring.

The remainder is organized as follows. Section 2 reviews computer vision-based cattle monitoring, lightweight object detection in livestock scenarios, and multi-scale feature fusion and attention mechanisms. Section 3 describes the dataset, the architecture of BoviFusionNet, and the details of ADown, BiFPN, and C2CGA. Section 4 presents the experimental setup, comparative study, ablation study, box-balanced augmentation results, visualization and error analysis, edge deployment on RK3588S, and discussion. Section 5 concludes this study.

## 2. Related Works

### 2.1. Computer Vision-Based Cattle Behavior Recognition

Automatic monitoring of cattle behavior has become an important research direction in precision livestock farming. Compared with manual observation and wearable sensors, computer vision-based methods offer a non-contact monitoring approach that can simultaneously record multiple animals and reduce interference with natural behaviors [19]. In cattle production, behaviors such as eating, standing, and lying are closely related to eating rhythm, comfort, welfare status, and potential health abnormalities. Consequently, accurate and continuous behavior recognition can provide valuable information for early warning and decision support in cattle health management.

Recent studies [20,21,22] have investigated visual cattle monitoring from various perspectives, including cattle detection, individual identification, behavior recognition, posture analysis, and tracking-based monitoring. Particularly, YOLO-based models have been used in this field because they offer a balance between detection accuracy and inference speed. Several existing works [23,24,25] have improved YOLO models by employing attention mechanisms, multi-scale feature fusion, lightweight convolutional modules, or enhanced feature extraction blocks to increase detection performance in complex farm environments. Their results demonstrated that deep learning-based visual monitoring is achievable for analyzing cattle behavior and facilitating automated monitoring.

Beyond computer vision, sensor-based approaches have also been widely used for monitoring cattle behavior. Wearable sensors such as tri-axial accelerometers, pedometers, rumen boluses, and Radio Frequency Identification (RFID) ear tags can provide continuous individual-level behavioral data, including eating duration, rumination time, lying bouts, and activity levels. These methods offer the advantage of unambiguous individual identification and long-term temporal monitoring without requiring a continuous visual line of sight. However, they require per-animal instrumentation, which can be costly at herd scale, may cause animal stress or discomfort, and involves ongoing maintenance (battery replacement, tag loss). In contrast, computer vision-based methods offer non-contact, multi-animal monitoring using fixed cameras without individual instrumentation, but face challenges in dense groupings, occlusion, and individual re-identification across camera views. The proposed BoviFusionNet is complementary to sensor-based approaches: vision-based behavior recognition can serve as a scalable initial screening tool, while sensor-based systems can provide finer-grained temporal and physiological data for animals flagged by the vision system.

Currently, practical cattle behavior recognition remains difficult in real barn and pasture environments. Cattle are often densely distributed, partially occluded, and captured at varying distances from the camera. Moreover, behavior categories usually exhibit subtle visual differences. For example, eating and standing share similar body postures except for head position and local contextual cues, while lying could be partially occluded by other animals or farm facilities. Hence, cattle behavior recognition requires not only accurate localization but also behavior-sensitive feature representation. Such challenges motivate the use of improved downsampling, multi-scale feature fusion, and attention-based contextual modeling in the proposed framework, which addresses essential design choices for translating AI-based behavior recognition into reliable tools for monitoring animals on farms.

### 2.2. Lightweight Object Detection in Livestock Scenarios

Object detection models have evolved from two-stage detectors to one-stage detectors and transformer-based detectors. Two-stage methods, such as Faster Region-based Convolutional Neural Networks (Faster R-CNN), offer good localization performance, but their generation and region-wise feature processing increase computational cost [26]. Transformer-based detectors, such as Detection Transformer (DETR) models and Real-Time-DETR (RT-DETR) variants, improve global feature modeling and end-to-end detection. Nevertheless, they also require higher computational resources for deployment optimization [27].

On the other side, YOLO-based one-stage detectors have been adopted in livestock applications due to their advanced inference speed. For instance, YOLOv5, YOLOv8, and YOLO11 offer different trade-offs between accuracy and efficiency and are employed as baseline models for edge-oriented detection tasks. Regarding cattle behavior recognition, lightweight YOLO models are valuable because farm-side devices usually have limited computing resources, memory capacity, and power budgets, constraints that are vital to deploying edge AI for real-time monitoring on working farms. However, lightweight detection is not equivalent to simply reducing the parameter count or GFLOPs. A compact model may lose discriminative feature representation, especially when behavior categories are visually similar or when targets are small and occluded. Therefore, lightweight livestock detectors must preserve useful posture and boundary information while remaining computationally efficient. To this end, the proposed method applies task-oriented structural improvements to a compact YOLO11-based detector, rather than simply scaling up model depth or width. It prioritizes feature quality for behavior-sensitive tasks and aligns with the need for practical, low-latency, and privacy-preserving edge AI solutions in this field.

### 2.3. Multi-Scale Feature Fusion and Attention Mechanisms

Multi-scale feature fusion is beneficial for object detection in complex livestock scenes. Particularly, in cattle-monitoring images, large foreground animals, distant cattle, and dense group targets often appear simultaneously [28]. Shallow features usually contain detailed spatial information useful for localization, whereas deep features carry stronger semantic information for behavior recognition. Conventional feature pyramid structures fuse these features through top-down or bottom-up pathways, but fixed fusion strategies may not fully adapt to varying target scales and scene conditions, a limitation that directly affects the reliability of behavior monitoring in farm environments.

BiFPN introduces learnable, normalized weights for feature fusion, enabling the network to adjust the contributions of different feature levels. This design is appropriate for cattle behavior recognition because the importance of spatial and semantic features varies with target size, degree of occlusion, and behavior category. So, in BoviFusionNet, BiFPN is adopted in the neck to enhance multi-scale representation while keeping the model compact. The implementation uses learnable fusion weights, followed by a Rectified Linear Unit (ReLU) activation and normalization before weighted feature summation, thereby supporting adaptive feature-level selection during training.

Attention mechanisms have also been employed to improve feature representation. For behavior recognition, attention can help the model focus on discriminative regions, such as the head, body contours, and local posture-related areas. Nevertheless, self-attention incurs high computational cost and memory overhead. For example, the EfficientViT model [29] by Liu et al. points out that actual inference speed is affected not only by parameter count or GFLOPs but also by memory access, tensor reshaping, element-wise operations, and operator efficiency. In this regard, efficient attention designs, such as cascaded group attention, aim to reduce redundancy among attention heads while improving attention diversity. So, BoviFusionNet uses C2CGA in the deep semantic stage. It is based on the Cross Stage Partial with Pyramid Squeeze Attention (C2PSA) architecture and replaces the attention branch with local window attention, which is appropriate for cattle behavior recognition because behavioral differences are often reflected in local posture and nearby context rather than in global image semantics alone. By focusing on behavior-sensitive local regions, the proposed method supports accurate detection.

In short, existing works have demonstrated the feasibility of computer vision-based cattle monitoring and lightweight object detection. However, limited object-level behavior balance, dense multi-scale cattle scenes, subtle inter-class differences, and edge-side deployment constraints remain insufficiently addressed. To bridge this research gap, we propose BoviFusionNet, which combines box-balanced learning, information-preserving downsampling, adaptive multi-scale fusion, local window attention, and RK3588S deployment validation for real-time cattle behavior recognition.

## 3. Materials and Methods

### 3.1. Dataset

This study uses a self-built dataset of cattle behavior, containing three categories: eating, standing, and lying, where each instance is labeled by its class and normalized bounding-box coordinates. These behaviors are closely related to eating activity, welfare, and daily management. Thus, their accurate recognition is fundamental to supporting assessments in precision livestock farming.

The self-built dataset was collected at a commercial dairy farm in Guangdong Province, China, housing approximately 500 lactating Chinese Holstein dairy cattle in a free-stall barn. Images were captured using the Hikvision DS-2CD2T47G2-L camera (4 megapixels) installed at a height of approximately 3.5 m with a top-down viewing angle. The original resolution was 2688 × 1520 pixels, downsampled to 640 × 640 pixels for model input. Videos were recorded at 15 frames per second (FPS) during October–December 2024, under natural daylight supplemented by barn Light Emitting Diode (LED) lighting. The weather during the collection period was predominantly clear to partly cloudy, with ambient temperatures ranging from 15 °C to 28 °C.

In detail, the dataset contains 1597 images: 1118 for training, 320 for validation, and 159 for testing. As shown in Table 1, the training split contains 5166 annotated boxes, consisting of 977 eating, 2469 standing, and 1720 lying instances. This distribution reveals a clear object-level imbalance, particularly for the eating class. Because cattle behavior recognition is an object detection task, effective training supervision depends on the number of annotated behavior instances rather than the number of images alone. Since one image may contain multiple cattle and several behavior categories simultaneously, image-level balancing cannot guarantee balanced learning for each behavior class. Such an imbalance could bias the model toward more frequent behaviors, compromising the reliability of health and welfare monitoring. To address this issue, an object-level box-balanced offline augmentation strategy was adopted. Unlike general image expansion, this strategy uses the number of annotated boxes as the primary balancing criterion. During augmentation, source images that help reduce the gap among behavior-instance counts are preferentially selected. Only the training split is augmented, while the validation and test splits remain unchanged, which increases the diversity and balance of training samples without contaminating the evaluation distribution, thereby supporting more equitable AI-based behavior recognition.

The dataset was split by video sequence rather than by random image selection. Videos were randomly assigned to the training, validation, and test sets in approximately 70%, 20%, and 10% splits. This sequence-level split prevents temporally adjacent frames from appearing in different splits, thereby avoiding inflated performance estimates from data leakage. Moreover, three annotators labeled the images using LabelImg, and a veterinary expert with experience in cattle practice reviewed all annotations. Inter-annotator agreement was evaluated using Cohen’s kappa, yielding a score of 0.91. Disagreements were resolved through consensus among annotators. Regarding behavior definitions, eating was defined as the head positioned at the feed bunk with visible eating activity (excluding grazing). Standing includes all four limbs supporting body weight with the head up. Lying includes both sternal and lateral recumbency. Head-down standing (without eating) is labeled as standing, not eating.

The augmentation operations include horizontal flipping, brightness and contrast perturbation, Hue-Saturation-Value (HSV) color perturbation, slight Gaussian blur, and slight noise. Vertical flipping, large-angle rotation, and aggressive cropping are not used because these operations generate unrealistic cattle postures or incomplete behavior patterns that could mislead health-related interpretations. After augmentation, the training split is expanded to 3000 images with 14,373 annotated boxes, including 4905 eating, 4905 standing, and 4563 lying instances. The final box-balanced dataset contains 3479 images and 16,602 annotated boxes, as listed in Table 2.

The source image selection strategy operated as follows. In each augmentation round, source images were selected from the training set such that, after augmentation, the gap between behavior-instance counts across classes was minimized. The selection was constrained so that each class could appear in at most a predefined maximum number of training images. After augmentation, eating instances increased from 977 to 4905 (+3928), standing from 2469 to 4905 (+2436), and lying from 1720 to 4563 (+2843). In terms of images containing each class (a single image may contain multiple classes), eating appeared in 1478 augmented training images, standing in 1106, and lying in 700.

We acknowledge that repeated animals and backgrounds from the same farm may increase the risk of overfitting. Thus, mitigation strategies include: (1) augmentation was applied only to the training set, not to the validation or test sets; (2) only mild augmentations were used (horizontal flip, brightness/contrast perturbation, HSV perturbation, slight Gaussian blur, slight noise). Vertical flip, large-angle rotation, and aggressive cropping were excluded because they would create unrealistic cattle postures; (3) all evaluation metrics are reported on an independent test set.

**Figure 1:**
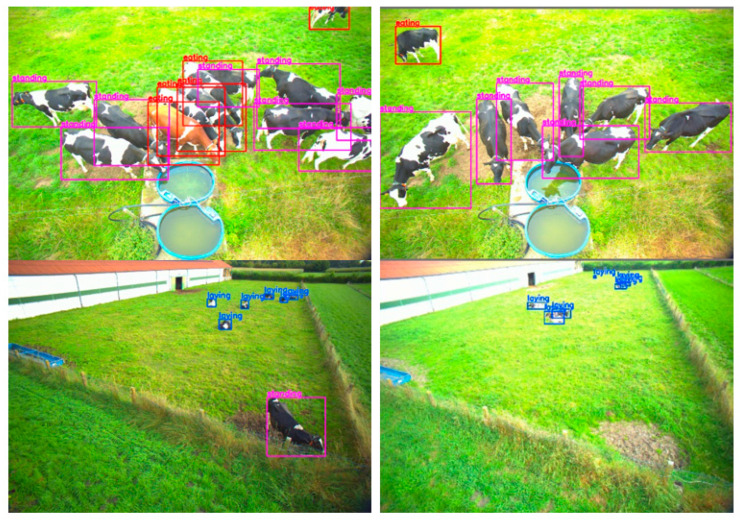
The box-balanced cattle behavior dataset primarily includes eating, standing, and lying behaviors.

Several samples from the self-built dataset are displayed in Figure 1, including dense cattle distributions, partial occlusion, distant targets, and large-scale variations. In addition, the visual differences among eating, standing, and lying behaviors can be subtle, especially when cattle overlap or appear far from the camera. Such characteristics make the dataset suitable for evaluating whether a detection model can preserve spatial details, fuse multi-scale features, and distinguish fine-grained behavior cues in the monitoring scenes.

### 3.2. Overall Architecture

**Figure 2:**
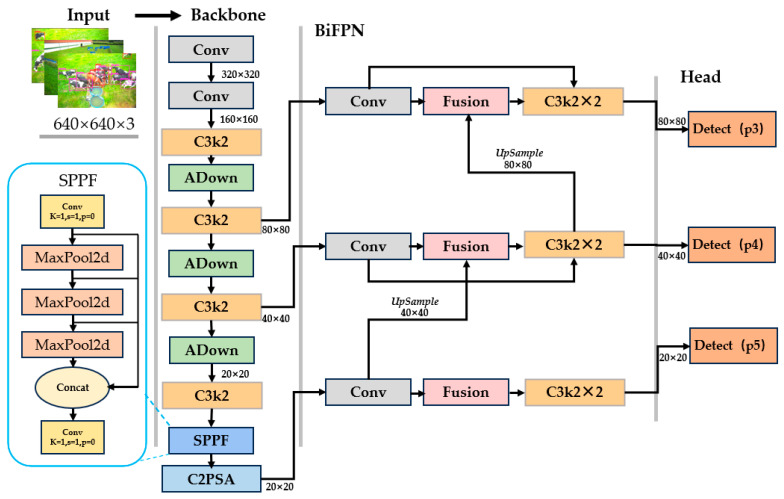
Overall architecture of the proposed BoviFusionNet for cattle behavior recognition.

BoviFusionNet is developed from a compact YOLO11-based one-stage detection architecture. As depicted in Figure 2, its overall architecture follows the typical backbone-neck-head paradigm, where the backbone extracts hierarchical visual features from the input image, the neck aggregates multi-scale features, and the detection head predicts bounding boxes and behavior categories at three feature scales. Given an input resolution of 640 × 640, the backbone progressively generates multi-level feature maps through convolutional blocks, ADown downsampling modules, Cross Stage Partial (C3k2) blocks, Spatial Pyramid Pooling-Fast (SPPF), and the proposed C2CGA attention module. These feature maps correspond to spatial resolutions of 80 × 80, 40 × 40, and 20 × 20, capturing cattle targets at different scales, from small distant individuals to large foreground animals. Since such scale variation is common in farm scenes, retaining multi-level features is essential for accurate behavior localization and detection.

Compared with the original YOLO11n, BoviFusionNet presents three main improvements to address cattle-specific challenges. First, ADown modules replace standard stride-based downsampling in the backbone to preserve local posture, body contour, and boundary information. Second, C2CGA is inserted after SPPF in the deep semantic stage to enhance local window contextual modeling, helping distinguish visually similar behaviors. Third, the neck adopts a BiFPN-style weighted fusion strategy that adaptively combines shallow spatial details with deep semantic features. Specifically, ADown is applied at the P3, P4, and P5 downsampling stages, C2CGA follows SPPF, and the neck employs repeated fusion nodes for bidirectional multi-scale aggregation. The neck first aligns channel dimensions of feature maps from three backbone stages via convolutional layers, then constructs top-down and bottom-up fusion paths. The top-down pathway upsamples high-level semantic features and fuses them with lower-level features to improve guidance for small and medium targets. Conversely, the bottom-up pathway progressively downsamples lower-level spatial features and fuses them with higher-level features to enhance localization and structural representation. This bidirectional process produces three enhanced feature maps, i.e., P3_out, P4_out, and P5_out, which are fed to the detection head. These components work together to benefit feature representation for dense, occluded, and multi-scale scenes of cattle behavior while keeping the model suitable for edge-oriented deployment.

### 3.3. Adaptive Downsampling (ADown)

**Figure 3:**
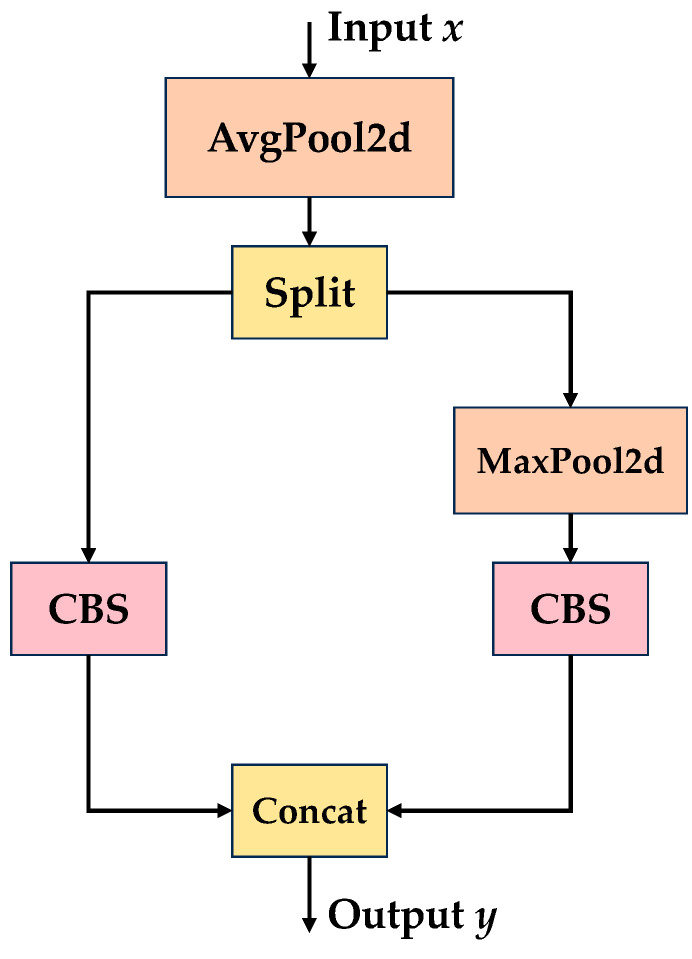
Structure of the ADown downsampling module.

Downsampling is necessary in object detection networks to reduce spatial resolution and increase receptive fields. For cattle behavior recognition, aggressive downsampling can weaken critical local cues, such as body contours, head position, limb posture, and boundaries between adjacent cattle [30]. These cues are vital for distinguishing visually similar behaviors and for detecting small or partially occluded cattle in dense scenes, which directly impacts the reliability of automated health and welfare monitoring. Therefore, an information-preserving downsampling is needed. To this end, BoviFusionNet replaces the standard stride-2 downsampling transitions in the backbone with the ADown module. As displayed in Figure 3, ADown employs a dual-branch structure that combines learnable convolutional downsampling with pooling-based context preservation. In the network configuration, ADown is introduced at the P3, P4, and P5 downsampling stages, facilitating the backbone to reduce feature-map resolution while retaining useful spatial and contextual information, an essential property for accurate behavior recognition under farm conditions.

Given an input feature map $X\in R^{C_{1}\times H\times W}$, ADown first applies an average-pooling operation to smooth local responses before downsampling:

(1) $$X^{\prime }=AvgPool\left(X\right)$$

Then, the pooled feature map is split into two channel groups:

(2) $$X_{1},X_{2}=Split\left(X^{\prime }\right)$$

The first branch applies a 3 × 3 convolution with stride 2 to learn discriminative downsampled features:

(3) $$Y_{1}={Conv}_{3\times 3,\;s=2}\left(X_{1}\right)$$

The second branch uses max pooling to retain salient local responses and applies a 1 × 1 convolution for channel projection:

(4) $$Y_{2}={Conv}_{1\times 1}\left(MaxPool\left(X_{2}\right)\right)$$

The outputs of the two branches are concatenated along the channel dimension:

(5) $$Y=Concat\left(Y_{1},{\;Y}_{2}\right)$$

In this study, ADown first applies average pooling, splits the feature map into two channel groups, processes one branch with a 3 × 3 stride-2 convolution, processes the other branch with max pooling followed by a 1 × 1 convolution, and then concatenates the two outputs. This design differs from a single stride-2 convolution by preserving complementary information from two paths: the convolution branch learns task-specific downsampled representations, while the pooling branch retains robust local responses and contextual cues.

For cattle behavior recognition, this dual-branch structure offers two advantages. One is that the convolutional branch learns discriminative patterns related to behavioral categories, such as posture and body orientation, which are essential for distinguishing healthy from abnormal behaviors. Another is that the pooling branch minimizes loss of salient local structures during resolution reduction, particularly for small targets and occluded cattle. Thus, by combining these branches, ADown provides an information-preserving transition for the backbone, improving feature quality without incurring significant computational overhead, enabling real-time behavior monitoring.

### 3.4. Bi-Directional Feature Pyramid Network (BiFPN)

Robust multi-scale feature representation is essential for cattle behavior recognition, as targets can appear as large foreground animals, medium-scale groups, or small distant objects within the same scene [31]. Shallow feature maps provide spatial details useful for locating small or occluded cattle, while deep feature maps carry stronger semantic information for behavior classification. Nonetheless, simple concatenation or unweighted summation treats all feature levels equally, failing to adapt to the varying importance of each scale. To address this, we apply a BiFPN-style weighted feature fusion strategy in the neck for adaptive multi-scale aggregation.

**Figure 4:**
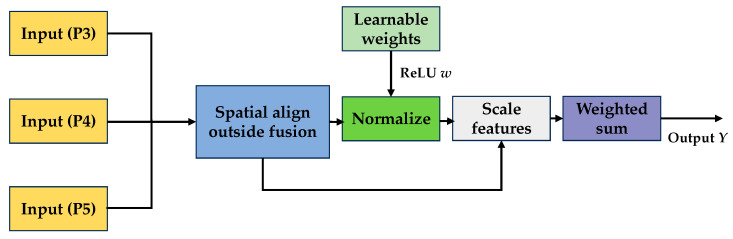
Structure of the BiFPN module.

As shown in Figure 4, the BiFPN module assigns a learnable weight to each input feature branch and normalizes these weights before summation. It enables the network to learn the relative contribution of different feature levels during training, a crucial capability for cattle behavior recognition, where high-resolution features benefit small or occluded targets and low-resolution semantic features aid behavior classification. Such adaptive fusion directly facilitates more accurate monitoring across diverse conditions.

Let the three backbone feature maps used by the neck be denoted as *C*_3_, *C*_4_, and *C*_5_, corresponding to feature resolutions of 80 × 80, 40 × 40, and 20 × 20, respectively. Before feature fusion, their channel dimensions are aligned to a unified channel number by a 1 × 1 convolution:

(6) $$P_{3}^{in}=\phi_{3}\left(C_{3}\right),{\;P}_{4}^{in}=\phi_{4}\left(C_{4}\right),\;P_{5}^{in}=\phi_{5}\left(C_{5}\right)$$

where $\phi_{i}\left(\cdot \right)$ denotes a channel-alignment convolution. In the proposed network, these aligned features are passed through a bidirectional fusion neck and generate $P_{3}^{out}$, $P_{4}^{out}$, and $P_{5}^{out}$ for three-scale detection.

For each fusion node, assume that there are *n* input feature maps $\left\{X_{1},{\;X}_{2},\;\ldots ,{\;X}_{n}\right\}$ with the same spatial resolution and channel dimension. A learnable scalar weight *w_i_* is assigned to each input branch. To ensure non-negative contribution and stable training, each weight is first constrained by ReLU:

(7) $$\;{\hat{w}}_{i}=ReLU\left(w_{i}\right),\;i=1,\;2,\;\ldots ,\;n$$

Subsequently, the non-negative weights are normalized by their sum:

(8) $$a_{i}=\frac{{\hat{w}}_{i}}{\sum_{j=1}^{n}{\hat{w}}_{i}+\varepsilon }$$

where $\varepsilon =10^{-4}$ is applied for numerical stability. The fused output of the node is computed as a weighted summation of all input features:

(9) $$Y=\sum_{i=1}^{n}a_{i}X_{i}$$

Compared with direct addition, this approach enables adaptive feature-level selection and prevents the network from treating all input branches equally. In addition, in the top-down fusion path, high-level semantic features are upsampled and fused with lower-level features. The P5 feature is upsampled and fused with $P_{4}^{in}$ to generate an intermediate P4 feature:

(10) $$P_{4}^{td}=F\left(Up\left(P_{5}^{in}\right),\;P_{4}^{in}\right)$$

where F(⋅) refers to the normalized weighted fusion operation, and Up(⋅) denotes nearest-neighbor upsampling.

Through this bidirectional weighted fusion strategy, BoviFusionNet adaptively integrates spatial details and semantic information across scales. For cattle behavior recognition, high-resolution features help separate adjacent cattle and locate small targets, while low-resolution features provide stronger category-level semantics. The learnable fusion weights help the BoviFusionNet to dynamically adjust the contributions of each scale, rather than relying on fixed fusion rules. As a result, the BiFPN neck improves the robustness of multi-scale behavior recognition in dense, occluded, and scale-varying cattle-monitoring scenes.

### 3.5. Cross-Channel Cross-Guided Attention (C2CGA)

Although multi-scale feature fusion enhances cattle localization across scales, accurate behavior recognition still depends on distinguishing fine-grained posture differences. As for cattle-monitoring scenes, eating, standing, and lying behaviors may share similar body textures and background conditions. Their differences are often reflected in local regions, such as head position, body contour, limb posture, and the spatial relationship between adjacent cattle. Hence, we design the C2CGA attention module in the deep semantic stage to advance local contextual representation and improve behavior-sensitive feature discrimination.

**Figure 5:**
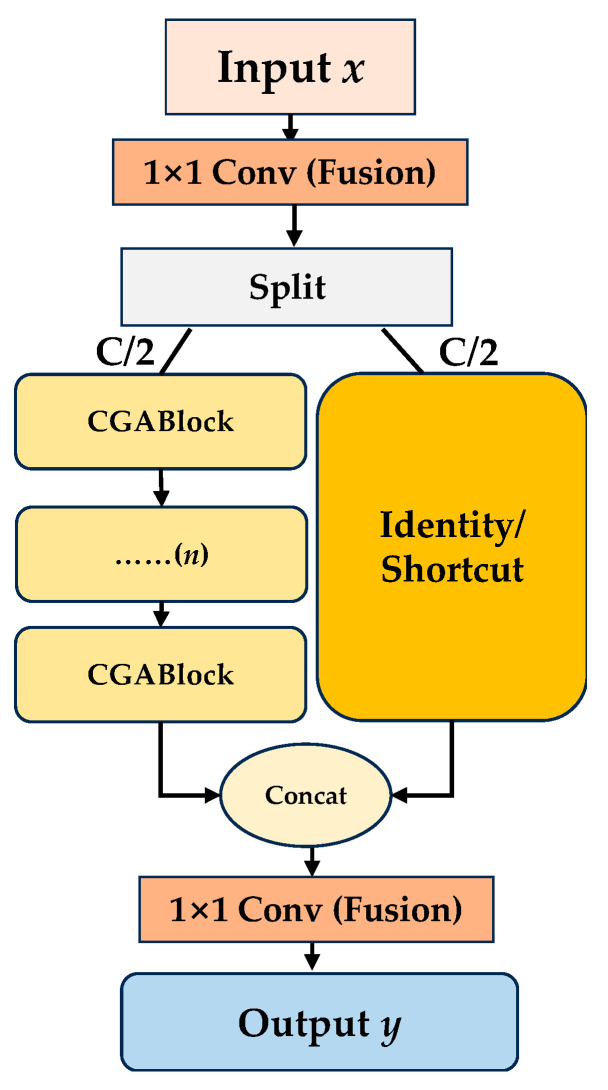
Structure of the C2CGA module.

As displayed in Figure 5, C2CGA follows a partial-attention structure. A 1 × 1 convolution first projects the input feature and then splits it into two-channel branches. Stacked Cascaded Group Attention (CGA) blocks process one branch for attention-based feature enhancement, while the other branch is retained as an identity shortcut to preserve the original semantic information. Finally, the two branches are concatenated and fused by another 1 × 1 convolution to generate the output feature. Here, C2CGA is built on the C2PSA structure, and its attention branch is replaced with CGABlock, which uses local window attention for local contextual modeling.

**Figure 6:**
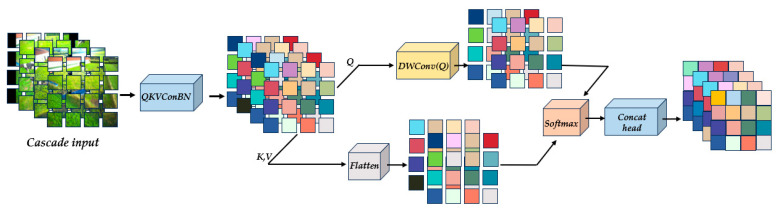
Cascaded group attention (CGA) in C2CGA.

Next, the internal cascaded group attention mechanism is drawn in Figure 6. For an input feature *U*, CGA generates Query *Q*, Key *K*, and Value *V* through a convolutional projection:

(11) Q,K,V=QKVConvBN(U)

To introduce local structural bias, a depthwise convolution is applied to the query feature *Q_l_*:

(12) $$Q_{l}=DWConv(Q)$$

Then, the attention matrix is computed from the query and key features:

(13) $$A=Softmax(\frac{Q_{l}K^{T}}{\sqrt{d}})$$

where *d* denotes the channel dimension of each attention head. The attention output is obtained by multiplying the attention matrix with the value feature:

(14) O = AV

In cascaded group attention, different attention heads are applied to process distinct feature groups rather than repeatedly attending to the same full feature representation, reducing attention redundancy and improving attention diversity, consistent with the motivation of cascaded group attention in EfficientViT, where EfficientViT shows that conventional attention may suffer from redundant attention maps and memory-inefficient operations [32], while cascaded group attention improves the efficiency–accuracy trade-off by feeding attention heads with different feature splits and cascading their outputs.

After all attention heads are computed, their outputs are concatenated and projected to form the final CGA output:

(15) $$CGA(U)=Proj(Concat(O_{1},\;O_{2},\;\ldots ,\;O_{h}))$$

where *h* refers to the number of attention heads.

Here, C2CGA is placed after the SPPF module in the deep semantic stage. This position is chosen because deep features carry stronger behavior-level semantic information, and applying attention at this stage enhances class-discriminative representations without increasing computation on high-resolution shallow features. For cattle behavior recognition, C2CGA helps the BoviFusionNet focus on local posture-related cues, such as the head-down pattern during eating, the body-support pattern during standing, and the horizontal body posture during lying. Therefore, this module improves the BoviFusionNet’s ability to distinguish visually similar behaviors in dense, occluded, and multi-scale scenes. Such fine-grained discrimination is essential for reliable automated monitoring of animals on farms.

### 3.6. Evaluation Metrics

Method performance was evaluated using precision, recall, F1-score, mAP@0.50, and mAP@0.50:0.95. Precision measures the proportion of correctly detected instances among all predicted instances, while recall measures the proportion of ground-truth instances that are successfully detected. Since cattle behavior recognition should reduce both false alarms and missed detections, the F1-score is used to measure the balance between precision and recall. In addition, mean Average Precision (mAP) is employed to evaluate overall localization and classification performance. In this study, mAP@0.50 refers to mAP calculated at an Intersection over Union (IoU) threshold of 0.50, while mAP@0.50:0.95 denotes the average mAP over IoU thresholds from 0.50 to 0.95 with a step size of 0.05. Furthermore, model complexity and deployment efficiency were evaluated using parameter count, GFLOPs, model size, and FPS.

(16) $$Precision=\frac{TP}{TP+FP}$$

(17) $$Recall=\frac{TP}{TP+FN}$$

where *TP*, *FP*, and *FN* denote true positives, false positives, and false negatives, respectively.

(18) $$F1=\frac{2\times Precision\times Recall}{Precision+Recall}$$

In addition, the mean average precision *AP_c_* is employed to evaluate the model’s overall localization and classification performance. For each class, it is calculated from the precision–recall curve:

(19) $$AP_{c}=\int_{0}^{1}P_{c}(R_{c})dR_{c}$$

where *P_c_* and *R_c_* refer to the precision and recall of class *c*, respectively. The final mean average precision over all *C* behavior categories is expressed as:

(20) $$mAP=\frac{1}{C}\sum_{c=1}^{C}AP_{c}$$

Furthermore, model complexity and deployment efficiency were also evaluated. The performance metrics include parameter count, GFLOPs, model size, and FPS. Parameter count and model size reflect storage requirements, while GFLOPs indicate computational complexity. FPS describes inference speed. Server-side FPS is used only as a relative efficiency indicator under the same software and hardware conditions, whereas practical edge-side speed is assessed via RK3588S INT8 deployment. The board-side deployment consists of Open Neural Network Exchange (ONNX) export, RKNN INT8 conversion, and RKNN Lite2 NPU inference, with an input size of 640 × 640. A consistent post-processing pipeline, i.e., YOLO raw-head decoding and class-wise Non-Maximum Suppression (NMS), is applied.

For RK3588S deployment, all compared models were converted to RKNN INT8 format using a 600-image calibration set, and the board-side benchmark measured only model inference time, excluding image saving and display. The confidence threshold, NMS IoU threshold, and IoU matching criterion were kept consistent across models, enabling us to evaluate not only server-side detection accuracy but also practical real-time feasibility on edge devices.

## 4. Results and Discussion

### 4.1. Experimental Setup

To ensure a fair comparison, all models were trained and evaluated under the same experimental setup. The box-balanced training split was used for model training, while the validation and test splits remained unchanged throughout all experiments, ensuring that reported performance improvements stem from enhanced training supervision and model architecture rather than from modifications to the evaluation set. The input image size was fixed at 640 × 640 for all models. Unless otherwise specified, each model was trained for 200 epochs with a batch size of 32. The optimizer was Stochastic Gradient Descent (SGD) with an initial learning rate of 0.01. Table 3 shows the hyperparameters.

### 4.2. Comparative Study

To evaluate BoviFusionNet’s overall performance, we compared it with mainstream models on the same dataset. The selected models contain lightweight YOLO-series (YOLOv5n, YOLOv8n, YOLO11n), larger YOLO-series (YOLOv5s, YOLOv8s), two-stage (Faster R-CNN), and transformer-based (RT-DETR), as presented in Table 4.

Compared with the YOLO11n baseline, BoviFusionNet improves recall from 0.7030 to 0.7851, F1-score from 0.7003 to 0.7763, mAP@0.50 from 0.7037 to 0.7976, and mAP@0.50:0.95 from 0.5313 to 0.6305. Meanwhile, the parameter count decreases from 2.58 M to 1.55 M, and the model size decreases from 5.2 MB to 3.4 MB. These results demonstrate that the proposed method enhances detection accuracy while preserving a compact model scale, a desirable trade-off for deploying AI-based monitoring on resource-limited edge devices.

Among the nano-scale YOLO models, YOLOv8n achieves a slightly higher mAP@0.50 than YOLO11n, but its mAP@0.50:0.95 falls below that of YOLOv5n and YOLO11n. YOLOv5n attains 0.5400 mAP@0.50:0.95, exceeding the YOLO11n baseline, yet its recall and mAP@0.50 remain lower than those of BoviFusionNet. Compared with these lightweight baselines, BoviFusionNet offers the best overall accuracy while using the lowest parameter count and the smallest model size. It can be said that the ADown, BiFPN, and C2CGA modules enhance feature representation more effectively than simply relying on the original nano-scale YOLO models.

Next, when compared with larger YOLO models, BoviFusionNet remains impressive. YOLOv8s achieves the highest recall among one-stage detectors (0.7923) and reaches 0.8001 mAP@0.50. However, it requires a parameter count of 9.83 M, 23.3 GFLOPs, and a model size of 19.0 MB. In contrast, BoviFusionNet offers a comparable mAP@0.50 of 0.7976 and a higher mAP@0.50:0.95 of 0.6305, with a parameter count of only 1.55 M, 5.4 GFLOPs, and a model size of 3.4 MB. Therefore, BoviFusionNet achieves detection quality comparable to or superior to that of other methods while maintaining substantially lower model complexity.

Moreover, Faster R-CNN yields the highest mAP@0.50 (0.8097) and mAP@0.50:0.95 (0.6345) among all compared methods. Nevertheless, this performance comes at the cost of extreme computational and storage demands: a parameter count of 41.36 M, 181.5 GFLOPs, and a model size of 158.1 MB. For edge-side cattle behavior recognition, such overhead is impractical. BoviFusionNet achieves a very close mAP@0.50:0.95 (only 0.004 lower) while reducing the parameter count by approximately 96.3% and GFLOPs by about 97.0%. This comparison further indicates a far more favorable trade-off between accuracy and complexity for edge AI deployment.

RT-DETR shows good F1-score performance (0.7733), but its mAP@0.50 and mAP@0.50:0.95 fall below those of BoviFusionNet. Meanwhile, it requires a parameter count of 9.48 M, 16.7 GFLOPs, and a model size of 18.4 MB, all of which are larger than those of the proposed model. It suggests that although transformer-based models possess greater representational capacity, they may not offer the remarkable efficiency for cattle behavior recognition, especially when targeting real-time monitoring on resource-constrained edge devices.

**Figure 7:**
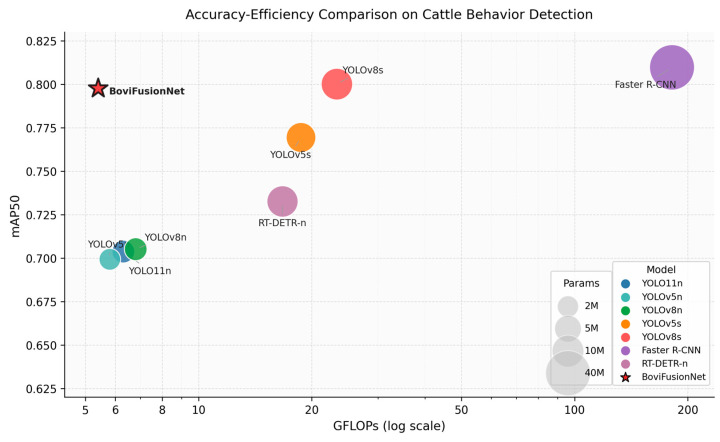
Accuracy–efficiency comparison of mainstream models on cattle behavior recognition, where the horizontal axis represents GFLOPs on a logarithmic scale, the vertical axis represents mAP@0.50, and the bubble size indicates parameter count.

To further illustrate the trade-off between detection accuracy and computational complexity, Figure 7 visualizes the results from Table 4. The horizontal axis represents GFLOPs on a logarithmic scale, the vertical axis represents mAP@0.50, and the bubble size indicates parameter count. Compared with mainstream models, BoviFusionNet lies in the upper-left region, demonstrating high detection accuracy with lower computational complexity and a lower parameter count. Although Faster R-CNN achieves the highest mAP@0.50, it requires substantially more GFLOPs and a higher parameter count. YOLOv8s also offers good accuracy, but its computational cost and model size are far higher than those of BoviFusionNet. These findings reveal that the proposed method offers a favorable trade-off between accuracy and efficiency for edge-oriented AI cattle behavior recognition.

Several recent YOLO-based methods for cattle behavior recognition have been proposed [33,34,35]. However, direct numerical comparison with these works is infeasible because they use different datasets with varying numbers of behavior categories, image resolutions, and evaluation protocols. Many of these studies do not release their datasets publicly. Therefore, we have discussed these methods qualitatively in Section 2 and ensured that all models compared in Table 4 were trained and evaluated under identical settings on our dataset, enabling a fair assessment of the proposed architectural improvements.

### 4.3. Ablation Study

To study the advances of the proposed method, a series of ablation experiments was conducted using YOLO11n. Please note that all experiments were performed under the same training and evaluation settings to ensure a fair comparison.

#### 4.3.1. Ablation Study of Different Proposed Modules

The first ablation experiment evaluates the individual and combined contributions of the three modules used in the BoviFusionNet, with the YOLO11n model trained on the box-balanced dataset serving as the baseline. The results are presented in Table 5, where ① means BiFPN, ② denotes C2CGA, and ③ refers to ADown.

From Table 5, introducing BiFPN alone increases mAP@0.50 from 0.7037 to 0.7506 and mAP@0.50:0.95 from 0.5313 to 0.5760, while reducing the parameter count from 2.58 M to 1.92 M. It indicates that adaptive multi-scale feature fusion handles small targets, dense cattle distributions, and large-scale variations without increasing model size. When C2CGA is used alone, mAP@0.50 rises to 0.7234 and mAP@0.50:0.95 to 0.5383, a smaller improvement than BiFPN, yet still demonstrating that local window attention contributes to behavior-sensitive feature representation. As C2CGA operates at the deep semantic stage, its primary benefit is fine-grained local contextual discrimination, which becomes even more valuable when combined with stronger multi-scale features for visually similar behaviors such as eating and standing. ADown alone increases mAP@0.50 to 0.7318 and mAP@0.50:0.95 to 0.5427, while reducing GFLOPs from 6.3 to 5.3, showing that ADown improves feature quality during downsampling and lowers theoretical complexity. Its dual-branch design preserves local information through pooling and convolution, which is especially beneficial for small or partially occluded cattle.

Regarding two-module combinations, BiFPN+C2CGA achieves 0.7775 mAP@0.50 and 0.5987 mAP@0.50:0.95, outperforming either module alone, indicating that adaptive fusion and local attention enhance different aspects of feature representation. BiFPN+ADown also yields the impressive two-module localization (0.7951 mAP@0.50 and 0.6193 mAP@0.50:0.95), indicating that information-preserving downsampling and adaptive fusion are highly complementary. C2CGA+ADown increases recall to 0.7635, suggesting that better-downsampled features enhance local attention effectiveness.

The BoviFusionNet, integrating all three modules, achieves the best overall performance, yielding gains of 9.39% in mAP@0.50 and 9.92% in mAP@0.50:0.95, while simultaneously reducing parameter count and model size. Hence, these results demonstrate that the three modules together provide a superior trade-off between accuracy and complexity for edge-oriented AI cattle behavior recognition.

#### 4.3.2. Ablation Study of Different Attention Modules

To validate the choice of C2CGA as the attention module, we compared several attention variants within the same YOLO11n. In this experiment, only the attention module in the deep semantic stage was replaced. The modules compared include original C2PSA (baseline), Cross Stage Boundary Refinement Attention (C2BRA), Cross Stage Partial Local Curvature-Guided Attention (C2LCGA), Cross Stage Partial Curvature-Guided Token Attention (C2CGTA), and the proposed C2CGA. This ablation experiment allows independent investigation of how different attention modules affect cattle behavior recognition.

As shown in Table 6, C2BRA and C2CGTA do not improve baseline performance, suggesting that more complex routing or attention structures are not suited to small-scale cattle behavior datasets. C2LCGA raises mAP@0.50 to 0.7156, but its recall and mAP@0.50:0.95 remain below baseline, indicating inconsistent benefit across evaluation metrics. Among all the modules compared, C2CGA achieves the best overall performance. These results demonstrate that C2CGA provides appropriate local contextual modeling for cattle behavior recognition, particularly for distinguishing visually similar behaviors in dense or partially occluded scenes, a key factor for reliable monitoring.

#### 4.3.3. Ablation Study of Different Downsampling Modules

Next, to evaluate the influence of different downsampling modules, we compared several typical methods under the same YOLO11n, including Alterable Kernel Convolution (AConv), Spatial-Channel Decoupled Downsampling (SCDown), Space-to-Depth Convolution (SPDConv), and ADown. Only the downsampling module was replaced, and all other components and training settings remained unchanged. This ablation study aims to identify the downsampling module well-suited for cattle behavior recognition, given that local posture cues, small targets, and occluded cattle are common.

From Table 7, SPDConv achieves the highest detection accuracy among the compared modules (0.7498 mAP@0.50 and 0.5638 mAP@0.50:0.95). However, it introduces a substantially larger computational burden: a parameter count of 4.59 M and 11.3 GFLOPs. Such complexity is less compatible with the edge-oriented design goal of this study. On the other side, ADown strikes a better balance between accuracy and efficiency. Compared with AConv and SCDown, ADown achieves higher recall, F1-score, and mAP@0.50 while maintaining low complexity. Its GFLOPs (5.3) are the lowest among the modules compared, and its model size remains compact at 4.3 MB. Although SPDConv offers higher accuracy, ADown aligns more closely with the lightweight and edge-deployment requirements of BoviFusionNet. These results demonstrate that for an edge-oriented AI system in cattle behavior recognition, the modules used should preserve local posture and boundary cues while keeping computational cost manageable. In this regard, ADown is well-suited, as it offers a trade-off between accuracy and complexity.

#### 4.3.4. Ablation Study of Different Feature Fusion Strategies

Furthermore, to assess the effectiveness of the BiFPN-based neck, we compared several feature-fusion variants under the same YOLO11n, including Asymptotic Feature Pyramid Network (AFPN), Hierarchical Scale-Based Feature Pyramid Network (HSFPN), context-guided FPN, Global Feature Pyramid Network (GFPN), and BiFPN. Only the neck’s fusion structure was replaced, with the backbone, detection head, dataset, and training settings remaining unchanged. This ablation study aims to identify a strategy that handles dense cattle distributions, small targets, and large-scale variations while maintaining a compact model size.

In Table 8, BiFPN achieves the best overall performance. Context-guided FPN also performs competitively, particularly in mAP@0.50:0.95, but requires a higher parameter count and a larger model size than BiFPN. HSFPN has lower complexity, yet its detection accuracy is lower than that of BiFPN. AFPN and GFPN do not yield stable improvements for this cattle behavior recognition task. These results demonstrate that BiFPN’s learnable weighted fusion mechanism is better suited to the multi-scale characteristics of cattle monitoring. By adaptively adjusting the contributions of different feature levels, BiFPN enhances the representation of small, occluded, and densely packed cattle targets without adding excessive model complexity, a key requirement in this study.

### 4.4. Box-Balanced Augmentation Results

To investigate the proposed box-balanced augmentation strategy, we trained YOLO11n on both the original and box-balanced training sets under the same experimental settings. This comparison isolates the effect of data-level improvement from model-structure modifications. Since the validation and test sets remained unchanged, performance differences primarily reflect the impact of the training-set distribution rather than shifts in the evaluation data.

The original training split contained 1118 images with 5166 annotated boxes (977 eating, 2469 standing, and 1720 lying instances), revealing a clear object-level class imbalance, particularly for the eating category. After box-balanced augmentation, the training split expanded to 3000 images with 14,373 annotated boxes (4905 eating, 4905 standing, and 4563 lying instances). Augmentation was applied only to the training split, while the validation and test splits were directly copied from the original dataset to maintain a clean evaluation protocol. The dataset construction also confirms that the annotations follow the YOLO format, with verified image-label consistency, valid class IDs, and normalized coordinates.

As shown in Table 9, using the box-balanced training set significantly improves the YOLO11n baseline without altering the network structure. Recall rises from 0.5966 to 0.7030, F1-score from 0.6434 to 0.7003, mAP@0.50 from 0.6451 to 0.7037, and mAP@0.50:0.95 from 0.4500 to 0.5313. Notably, mAP@0.50:0.95 improves by 8.13%, indicating that object-level balancing enhances both detection sensitivity and localization quality under stricter IoU thresholds.

**Figure 8:**
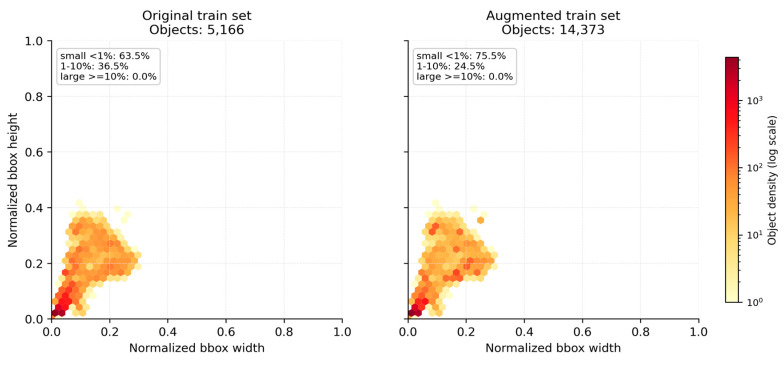
Comparison of normalized bounding-box size distributions in the original and box-balanced training sets. The horizontal axis represents normalized bounding-box width, and the vertical axis represents normalized bounding-box height. The augmented training set increases sample density while largely preserving the original target-scale distribution.

To examine whether augmentation alters target-scale characteristics, Figure 8 compares the normalized distributions of bounding-box width and height between the original and augmented training sets. The original set contains 5166 boxes, while the augmented set contains 14,373 boxes. Although the number of instances increases substantially, the overall distribution pattern remains similar: most instances are small targets, medium-scale targets account for the remainder, and large targets are nearly absent. It indicates that the proposed augmentation primarily supplements valid training samples rather than introducing abnormal annotations or artificially distorted scale distributions. This property is vital because aggressive augmentation can generate unrealistic object sizes, incomplete cattle bodies, or invalid behavior patterns, leading to biased evaluation. In contrast, our strategy increases object-level class balance while preserving the dataset’s original scale characteristics.

Overall, the box-balanced augmentation strategy provides a more suitable training basis for cattle behavior recognition. It expands annotated behavior instances, alleviates object-level imbalance, and maintains realistic target-scale distributions, supporting the development of equitable AI models for automated monitoring on farms. Therefore, the improvements shown in Table 9 can be reliably attributed to increased sample diversity and balanced supervision across eating, standing, and lying behaviors, rather than to artificial changes in the evaluation distribution or to abnormal target-size generation.

### 4.5. Visualization and Error Analysis

To provide an intuitive understanding of model performance beyond the numerical metrics reported above, we conducted a visualization analysis from four perspectives: class-level confusion, precision–recall characteristics, feature response regions, and qualitative detection results.

**Figure 9:**
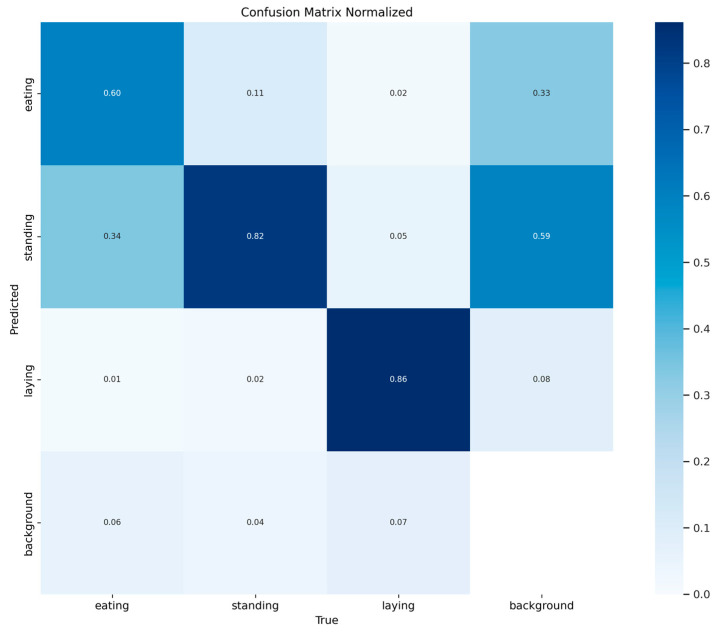
Normalized confusion matrix of BoviFusionNet on cattle behavior recognition. The matrix shows that standing and lying are recognized more reliably, while eating is more likely to be confused with standing.

Figure 9 presents the normalized confusion matrix of BoviFusionNet. The diagonal values indicate the proportion of correctly classified instances for each behavior category. The model achieves relatively high correct classification ratios for standing (0.82) and lying (0.86), indicating that these two behaviors are recognized more reliably. In contrast, the diagonal value for eating is only 0.60, notably lower than the other two classes. The primary confusion occurs between eating and standing: 0.34 of true eating instances are predicted as standing, while 0.11 of true standing instances are predicted as eating. It aligns with the visual characteristics of cattle behavior. Eating and standing cattle often share similar body appearances, with differences mainly reflected in local cues such as head position, eating direction, and surrounding context. When the head region is partially occluded, or the target is small, the model may mistake standing for eating. Lying, by comparison, exhibits a more distinct horizontal body posture, which explains its higher accuracy. Such confusion highlights the need for fine-grained behavioral discrimination to support reliable monitoring.

Table 10 presents the per-class precision, recall, F1-score, AP50, and AP50-95 for the three behavior categories. Eating achieves the lowest performance across all metrics (AP50 = 0.6005, AP50-95 = 0.4638), lower than standing (AP50 = 0.8556) and lying (AP50 = 0.9367). The results reveal that eating is the most challenging category due to its visual similarity to standing, as both behaviors share similar body postures, except for subtle head position and local eating-context cues. Such per-class results directly motivate the C2CGA module’s local window attention, which targets fine-grained discrimination within behavior-sensitive local regions.

**Figure 10:**
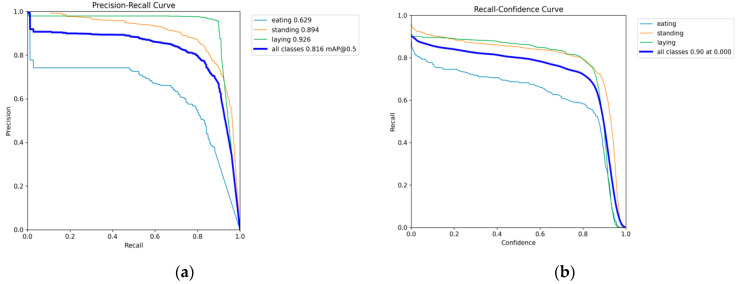
Precision–recall and recall–confidence curves of BoviFusionNet: (**a**) precision–recall curve; (**b**) recall–confidence curve.

Figure 10 shows the precision–recall and recall–confidence curves of BoviFusionNet. From Figure 10a, the model achieves an overall mAP@0.50 of 0.816 across all behavior categories. Among the three classes, lying yields the highest AP value (0.926), followed by standing (0.894), while eating yields the lowest AP value (0.629). This class-wise trend aligns with the confusion matrix, confirming that eating remains the most challenging category in this dataset, an important consideration for developing balanced behavior-monitoring systems.

Figure 10b shows the recall–confidence curve. Overall recall remains relatively stable at low and medium confidence thresholds but drops rapidly when the threshold approaches a high value. This trend implies that several valid detections, especially small or visually ambiguous targets, may have moderate confidence scores. Therefore, in practical deployment, the confidence threshold should be selected based on the application priority: a lower threshold reduces missed detections, whereas a higher threshold minimizes false alarms.

**Figure 11:**
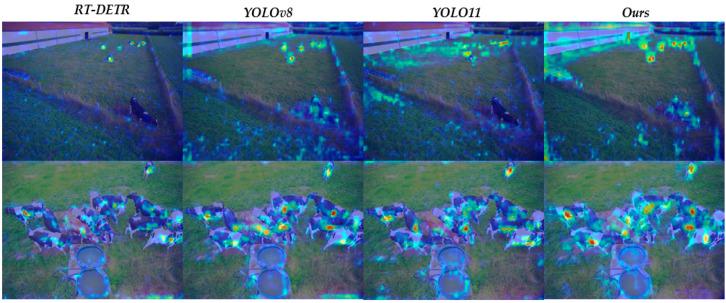
Heatmap visualization comparison among RT-DETR, YOLOv8, YOLO11, and BoviFusionNet. Warmer regions indicate areas that contribute more strongly to model detection.

**Figure 12:**
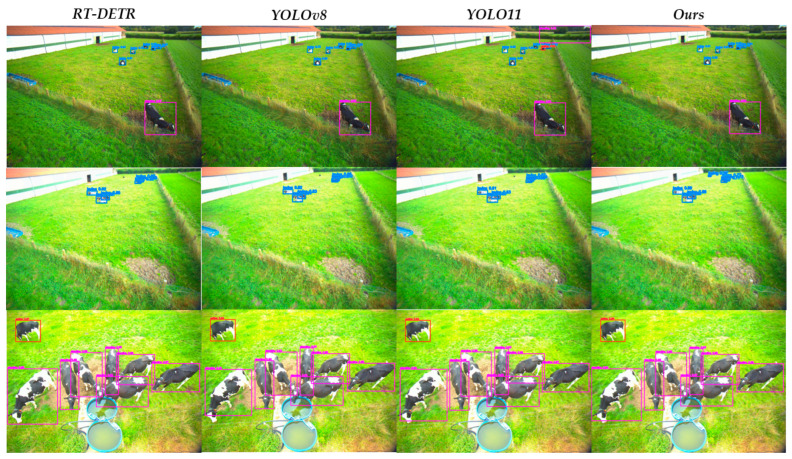
Qualitative detection comparison among RT-DETR, YOLOv8, YOLO11, and BoviFusionNet on cattle behavior images, including small distant lying cattle, dense groups of standing and eating cattle, and partially occluded targets.

Subsequently, to better understand the feature responses of different models, we used a heatmap to compare RT-DETR, YOLOv8, YOLO11, and BoviFusionNet. As illustrated in Figure 11, these models exhibit different attention distributions in cattle behavior scenes. Compared with the others, BoviFusionNet shows more concentrated responses on cattle body regions and behavior-related local areas. In dense scenes, the proposed model focuses more clearly on cattle groups rather than background grassland. In small-target scenes, it produces more noticeable activation around distant cattle instances. Meanwhile, Figure 12 presents qualitative detection results of the same models on representative cattle behavior images, including small distant lying cattle, dense groups of standing and eating cattle, and partially occluded targets. These scenes are challenging due to large-scale variations and the small image area occupied by several instances.

Based on these results, BoviFusionNet demonstrates stable performance in complex scenes. For small distant targets, it better preserves weak target cues and reduces missed detections. For dense groups, it maintains clearer localization of adjacent cattle and provides reliable predictions of cattle behavior. In multi-class scenes that include both eating and standing behaviors, BoviFusionNet also performs competitively, though several confusions persist due to similar body postures and local occlusions. This qualitative comparison is consistent with the quantitative results in Section 4.2 and the ablation study in Section 4.3.

Overall, the confusion matrix and precision–recall curves show that lying and standing are detected more reliably, while eating remains the most difficult behavior category. The heatmaps reveal that the proposed method focuses more effectively on cattle-related regions and local behavior cues, while the qualitative detection results further confirm that BoviFusionNet is robust in dense, occluded, and multi-scale scenes. These findings indicate that combining information-preserving downsampling, adaptive multi-scale fusion, and local window attention enhances the visual representation required for cattle behavior recognition, an essential step toward reliable edge AI-based detection in farm management.

### 4.6. Edge Deployment on RK3588S

To assess the practical feasibility of BoviFusionNet’s deployment, we conducted edge-side inference experiments on an RK3588S device. The trained PyTorch model was first exported to ONNX format and then converted to RKNN INT8 format using the RKNN Toolkit. During board-side inference, RKNN Lite2 executed the model on the RK3588S NPU. The input image size was fixed at 640 × 640, and the post-processing pipeline included YOLO raw-head decoding, Distribution Focal Loss (DFL) decoding, and class-wise non-maximum suppression [36].

**Figure 13:**
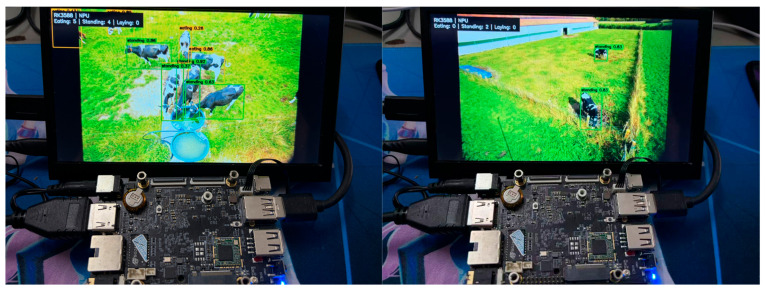
Edge deployment of the proposed BoviFusionNet on an RK3588S device for board-side cattle behavior recognition across various images.

All compared models were converted to YOLO raw-head RKNN INT8 format and calibrated using the same 600-image calibration set. The FPS values reported in this section measure only model inference time, excluding image saving and display. The confidence threshold, NMS IoU threshold, and matching IoU threshold were kept consistent across models. This standardized protocol ensures a fair comparison of edge-side performance, which is vital for deploying systems on resource-limited devices. Figure 13 shows the RK3588S used for board-side cattle behavior recognition, in which the deployed INT8 RKNN model identifies behavior instances across various images.

Table 11 compares the FP32 (CPU ONNX) and INT8 (NPU RKNN) inference results on the same validation subset. The INT8 quantization incurs negligible accuracy loss across all models: for BoviFusionNet, mAP@0.50 decreases from 0.790 to 0.781 (−0.009) and mAP@0.50:0.95 from 0.626 to 0.617 (−0.009), while the model size is reduced by 73.5% (from 3.4 MB to 0.9 MB) and NPU inference achieves a 3.20× speedup over CPU. BoviFusionNet achieves 28.08 FPS on the RK3588S NPU, satisfying the practical real-time requirement for cattle behavior recognition. Table 12 further reports the end-to-end deployment latency on the RK3588S device, including preprocessing (image resizing and normalization), NPU inference, and postprocessing (YOLO raw-head decoding, DFL decoding, and class-wise NMS). BoviFusionNet achieves an end-to-end latency of 42.96 ms (23.28 FPS), with a 95% confidence interval of 42.18–43.22 ms.

Regarding edge device resource consumption, the RK3588S draws approximately 7–10 W during NPU inference (measured at the board level with a USB power meter). The NPU utilization reaches approximately 85–90% during continuous inference, and peak memory usage is approximately 1.2 GB, including the RKNN model, input/output buffers, and the RKNN Lite2 runtime. Under ambient temperatures of 20–25 °C, continuous inference over 24 h showed stable FPS with no observable thermal throttling. Formal thermal stress testing across a wider temperature range (e.g., 0–40 °C) and detailed power profiling under different workloads are reserved for future work.

An explanation is that standard YOLO models primarily consist of conventional convolution, normalization, activation, upsampling, and detection-head operations, all of which are well optimized by embedded inference libraries and NPU runtimes [37]. In contrast, BoviFusionNet employs BiFPN-style weighted fusion and C2CGA attention, which involve multi-branch fusion, normalized weighted summation, local window attention, tensor reshaping, and additional feature-map movement. Such operations can increase memory access and reduce the efficiency of operator execution on the RK3588S NPU, even when parameter count and GFLOPs are lower. It reflects the gap between theoretical complexity and the efficiency of hardware-aware inference. The proposed model still reaches 28.08 FPS, satisfying real-time monitoring requirements for on-farm assessment. Future work should focus on NPU-oriented optimization, including simplifying fusion nodes, replacing attention operations with more hardware-friendly designs, and enhancing RKNN graph-level operator fusion.

### 4.7. Discussion

First, the experimental results demonstrate that the proposed task-specific integration of ADown, BiFPN, and C2CGA successfully achieves the study’s objective: BoviFusionNet improves mAP@0.50:0.95 by 9.92% over the YOLO11n baseline, reduces the parameter count by 39.8%, and achieves 28.08 FPS on the RK3588S edge device. This accuracy–efficiency trade-off confirms that high-performance cattle behavior recognition is feasible on resource-limited devices without relying on cloud services or wearable sensors. The proposed method achieves reliable recognition of eating, standing, and lying behaviors under dense, occluded, and multi-scale farm conditions. Behavior recognition is only an intermediate step toward automated health and welfare assessment. For instance, reduced eating frequency or prolonged lying may signal lameness, illness, or thermal stress, yet such inferences require temporal pattern analysis across consecutive frames. The box-balanced learning strategy employed in this study provides an equitable representation of each behavior, reducing the risk of behavior-level bias that could affect subsequent health-related analyses. Future work will integrate sequential models on BoviFusionNet’s frame-wise detections to capture behavioral rhythms and detect deviations indicative of compromised welfare. However, we emphasize that these behavioral changes are suggestive rather than diagnostic, as formal welfare assessment requires validated scoring protocols and veterinary clinical examination.

Second, our RK3588S deployment results reveal a gap between server-side metrics and actual edge inference speed. While BoviFusionNet reduces complexity by 39.8% relative to YOLO11n, its on-board FPS (28.08) is lower than that of several YOLO models. This finding highlights that lightweight architecture design must go beyond FLOP reduction. NPU-friendly operators, minimal tensor reshaping, and reduced memory access patterns are equally critical. The BiFPN-style weighted fusion and C2CGA attention, though effective for accuracy, present multi-branch operations that current NPU compilers do not fully optimize. Therefore, we advocate co-design between detection algorithms and edge devices by simplifying fusion nodes, replacing attention with hardware-efficient alternatives, and leveraging graph-level operator fusion.

Third, several limitations should be acknowledged. The dataset, while box-balanced, originates from a single farm. Cross-farm and seasonal variations in lighting, background, and cattle breeds may affect generalization. In addition, the proposed method operates on individual images without temporal memory, which limits its ability to detect transient abnormal behaviors or predict high-level health events. Hence, future work will prioritize two directions. On the one hand, we will expand data diversity by collecting multi-farm, multi-season cattle behavior datasets and incorporating lightweight temporal modules that incur minimal latency. On the other hand, we will pursue hardware-aware neural architecture search tailored for NPU deployment, alongside integrating on-edge alert systems that notify farmers of early health risks in real time.

Next, regarding practical deployment, the current validation was conducted on approximately 500 dairy cattle. The lightweight architecture (1.55 M parameters, 3.4 MB model size) and edge-deployment capability make the system economically scalable: multiple cameras can be connected to the RK3588S devices, each processing one or two video streams at real-time speeds. Larger herds would require multiple edge devices, which is feasible given the low per-unit hardware cost of the RK3588S device. Concerning environmental applicability, the self-built dataset and validation were conducted in a free-stall dairy barn environment with adjacent outdoor exercise areas and eating alleys. The reported performance is directly applicable to indoor barn monitoring scenarios. Deployment in paddock or pasture environments presents additional challenges, including longer camera-to-animal distances, more severe occlusion from vegetation and topography, highly variable natural lighting conditions (direct sunlight, deep shadows), and adverse weather (rain, fog, wind). While the proposed BiFPN multi-scale fusion and ADown information-preserving downsampling partially address scale variation and low-resolution targets, we have not yet systematically validated BoviFusionNet across diverse outdoor grazing systems with varying vegetation, terrain, and weather conditions. We identify cross-environment generalization, from the current free-stall setting to more diverse outdoor grazing and pasture systems, as an important direction for future work, one that requires multi-environment data collection and domain adaptation techniques.

Furthermore, the current system provides frame-level behavior recognition as a foundational component. The veterinary relevance of this work could be substantially enhanced by integrating the detected behavioral metrics (e.g., daily eating duration, lying time, standing/lying ratio) with established veterinary health records, such as lameness scores, Body Condition Scores (BCS), feed intake data, rumination time, and clinical disease records. Such multimodal integration would enable more direct inferences about health and welfare and strengthen the connection to veterinary science.

Finally, we note that the proposed vision-based system is complementary to, rather than a replacement for, sensor-based monitoring approaches (e.g., accelerometers, rumen boluses). Vision-based detection provides scalable, non-contact screening across the herd, while sensor-based methods can offer finer-grained individual-level temporal data (e.g., rumination time, lying bout duration) for animals identified as requiring closer attention. Future work could explore multimodal fusion of camera-based behavior recognition with wearable sensor data to combine the scalability of vision with the temporal precision of sensors.

## 5. Conclusions

This study proposed BoviFusionNet, a lightweight edge-oriented AI system that enables real-time, non-contact monitoring of cattle eating, standing, and lying behaviors. To mitigate object-level class imbalance, a key obstacle in livestock behavior analysis, we adopted a box-balanced augmentation strategy that rebalances training instances without altering evaluation distributions. Complemented by three vital modules (ADown, BiFPN, and C2CGA), BoviFusionNet achieves a favorable accuracy-complexity trade-off. It improves mAP@0.50:0.95 by 9.92% over YOLO11n while reducing parameter count by 39.8%. Furthermore, we deployed BoviFusionNet on the RK3588S edge device using INT8 quantization, achieving 28.08 FPS, which is sufficient for real-time on-farm monitoring and farm management. These results demonstrate that high-performance cattle behavior recognition is achievable on resource-limited hardware, a prerequisite for scalable, privacy-preserving precision livestock farming.

By enabling continuous, automated recognition of health-relevant behaviors, this study provides a practical foundation for enhancing automated livestock behavior monitoring, assisting farm management decision-making, and providing behavioral cues that, when combined with temporal behavior analysis, may ultimately contribute to the early detection of health disorders. Crucially, our results show that task-specific architectural balance and data-level fairness shift the paradigm from simply chasing benchmark metrics to designing fit-for-purpose AI that respects the real-world constraints of livestock environments, where interpretability, low latency, and equitable representation of behavior are as important as raw performance. Future extensions will focus on cross-farm generalization, temporal behavior modeling, and further hardware-aware optimization to transition from behavior recognition to actionable health management. Meanwhile, deep learning or machine learning methods in related fields [38,39,40,41,42,43] will also be referenced to optimize the deployment model.

## Figures and Tables

**Table 1:** Details of the box-balanced cattle behavior dataset before augmentation.

Dataset	Images	Eating	Standing	Lying	Boxes
Train	1118	977	2469	1720	5166
Validation	320	252	721	500	1473
Test	159	143	343	270	756
All	1597	1372	3533	2490	7395

**Table 2:** Details of the box-balanced cattle behavior dataset after augmentation.

Dataset	Images	Eating	Standing	Lying	Boxes
Train	3000	4905	4905	4563	14,373
Validation	320	252	721	500	1473
Test	159	143	343	270	756
All	3479	5300	5969	5333	16,602

**Table 3:** The hyperparameters for training BoviFusionNet.

Hyperparameter	Detail
Input Size	640 × 640
Epoch	200
Initial Learning	0.01
Workers	8
Batch Size	32
Optimizer	Stochastic Gradient Descent (SGD)
Environment	Linux, Python 3.10.18, PyTorch 2.2.1, CUDA 11.8

**Table 4:** Performance comparison of BoviFusionNet with mainstream models.

Model	Recall	F1-Score	mAP@0.50	mAP@0.50:0.95	Parameters (M)	GFLOPs	Model Size
YOLOv5n	0.6863	0.7178	0.6993	0.5400	2.18	5.8	4.5
YOLOv8n	0.6978	0.7192	0.7052	0.4994	2.68	6.8	5.4
YOLO11n	0.7030	0.7003	0.7037	0.5313	2.58	6.3	5.2
YOLOv5s	0.7681	0.7559	0.7694	0.6093	7.81	18.7	15.2
YOLOv8s	0.7923	0.7637	0.8001	0.6216	9.83	23.3	19.0
Faster R-CNN	0.7716	0.7885	0.8097	0.6345	41.36	181.5	158.1
RT-DETR	0.7521	0.7733	0.7326	0.5422	9.48	16.7	18.4
BoviFusionNet	0.7851	0.7763	0.7976	0.6305	1.55	5.4	3.4

**Table 5:** Ablation study of the three modules (BiFPN, C2CGA, and ADown) in BoviFusionNet.

①	②	③	Recall	F1-Score	mAP@0.50	mAP@0.50:0.95	Parameters (M)	GFLOPs	Model Size
×	×	×	0.7030	0.7003	0.7037	0.5313	2.58	6.3	5.2
√	×	×	0.7383	0.7514	0.7506	0.5760	1.92	6.3	4.0
×	√	×	0.7215	0.7066	0.7234	0.5383	2.56	6.3	5.3
×	×	√	0.7173	0.7219	0.7318	0.5427	2.10	5.3	4.3
√	√	×	0.7551	0.7600	0.7775	0.5987	1.90	6.3	4.0
√	×	√	0.7497	0.7758	0.7951	0.6193	1.58	5.4	3.4
×	√	√	0.7635	0.7679	0.7891	0.5786	2.08	5.3	4.4
√	√	√	0.7851	0.7763	0.7976	0.6305	1.55	5.4	3.4

**Table 6:** Ablation study of different attention modules.

Module	Recall	F1-Score	mAP@0.50	mAP@0.50:0.95	Parameters (M)	GFLOPs	Model Size
C2PSA	0.7030	0.7003	0.7037	0.5313	2.58	6.3	5.2
C2BRA	0.6796	0.6757	0.6914	0.5156	2.60	6.3	5.3
C2LCGA	0.6564	0.6739	0.7156	0.5226	1.82	6.3	5.3
C2CGTA	0.6350	0.6534	0.6884	0.5140	2.74	6.3	5.3
C2CGA	0.7215	0.7066	0.7234	0.5383	2.56	6.3	5.3

**Table 7:** Ablation study of different downsampling modules.

Module	Recall	F1-Score	mAP@0.50	mAP@0.50:0.95	Parameters (M)	GFLOPs	Model Size
AConv	0.6782	0.7153	0.7162	0.5395	2.58	6.3	5.2
SCDown	0.5814	0.5832	0.6118	0.5456	2.00	5.5	4.2
SPDConv	0.7229	0.7317	0.7498	0.5638	4.59	11.3	9.1
ADown	0.7173	0.7219	0.7318	0.5427	2.10	5.3	4.3

**Table 8:** Ablation study of different feature fusion strategies.

Method	Recall	F1-Score	mAP@0.50	mAP@0.50:0.95	Parameters (M)	GFLOPs	Model Size
AFPN	0.5794	0.5278	0.5819	0.4077	2.66	8.8	5.5
HSFPN	0.6926	0.7003	0.7081	0.4943	1.82	5.6	3.8
Context-Guided FPN	0.7256	0.7390	0.7423	0.5726	2.74	6.5	5.6
GFPN	0.5787	0.6374	0.6658	0.4812	3.66	8.2	7.5
BiFPN	0.7383	0.7514	0.7506	0.5760	1.92	6.3	4.0

**Table 9:** Effect of box-balanced augmentation on YOLO11n.

Model	Recall	F1-Score	mAP@0.50	mAP@0.50:0.95	Parameters (M)	GFLOPs	Model Size
YOLO11n (without augmentation)	0.5966	0.6434	0.6451	0.4500	2.58	6.3	5.3
YOLO11n (with augmentation)	0.7030	0.7003	0.7037	0.5313	2.58	6.3	5.2

**Table 10:** Per-class detection performance of BoviFusionNet on the cattle behavior test set.

Class	Precision	Recall	F1-Score	mAP@0.50	mAP@0.50:0.95
Eating	0.5869	0.6825	0.6311	0.6005	0.4638
Standing	0.7649	0.8086	0.7862	0.8556	0.6766
Lying	0.9643	0.8643	0.9116	0.9367	0.7510
All	0.7720	0.7851	0.7763	0.7976	0.6305

**Table 11:** CPU ONNX and RKNN NPU INT8 deployment comparison on the RK3588S device.

Model	Format	Model Size (MB)	Size Reduction	Precision	Recall	F1-Score	mAP@0.50	mAP@0.50:0.95	FPS (RK3588S)	Speedup
YOLOv5n	FP32	4.5	—	0.780	0.808	0.794	0.689	0.530	9.75 (CPU)	—
	INT8	1.2	73.3%	0.788	0.804	0.796	0.675	0.521	39.34 (NPU)	4.03×
YOLOv8n	FP32	5.4	—	0.790	0.777	0.783	0.700	0.488	8.72 (CPU)	—
	INT8	1.4	74.1%	0.794	0.781	0.787	0.692	0.473	50.67 (NPU)	5.81×
YOLO11n	FP32	5.2	—	0.812	0.796	0.804	0.695	0.527	8.63 (CPU)	—
	INT8	1.4	73.1%	0.815	0.788	0.801	0.689	0.519	34.56 (NPU)	4.00×
BoviFusionNet	FP32	3.4	—	0.877	0.834	0.855	0.790	0.626	8.76 (CPU)	—
	INT8	0.9	73.5%	0.874	0.837	0.855	0.781	0.617	28.08 (NPU)	3.20×

**Table 12:** INT8 RKNN-NPU end-to-end deployment performance on the RK3588S device.

Model	Preprocessing (ms)	NPU Inference (ms)	Postprocessing (ms)	End-to-End Latency (ms)	95% CI (ms)	End-to-End FPS
YOLOv5n	3.12	18.84	6.00	27.96	27.23–28.46	35.77
YOLOv8n	3.05	14.23	5.10	22.38	21.88–22.59	44.68
YOLO11n	3.45	22.01	7.20	32.66	32.32–33.27	30.62
BoviFusionNet	3.78	31.02	8.16	42.96	42.18–43.22	23.28

## Data Availability

The datasets generated and/or analyzed during the current study are available at https://github.com/AHAPPYMAN-666/BoviFusionNet (accessed on 13 June 2026).
